# Resveratrol and Resveratrol-Loaded Galactosylated Liposomes: Anti-Adherence and Cell Wall Damage Effects on *Staphylococcus aureus* and MRSA

**DOI:** 10.3390/biom13121794

**Published:** 2023-12-14

**Authors:** Giuliana Prevete, Beatrice Simonis, Marco Mazzonna, Francesca Mariani, Enrica Donati, Simona Sennato, Francesca Ceccacci, Cecilia Bombelli

**Affiliations:** 1 Department of Chemistry and Technology of Drug, Sapienza University of Rome, Piazzale Aldo Moro 5, 00185 Rome, Italy; giuliana.prevete@uniroma1.it; 2 Institute for Biological Systems of Italian National Research Council (ISB-CNR), Area della Ricerca di Roma 1, Via Salaria Km 29,300, 00015 Monterotondo, Italy; enrica.donati@cnr.it; 3 Institute for Biological Systems of Italian National Research Council (ISB-CNR), Secondary Office of Rome-Reaction Mechanisms c/o Department of Chemistry, Sapienza University of Rome, Piazzale Aldo Moro 5, 00185 Rome, Italyfrancesca.ceccacci@cnr.it (F.C.); cecilia.bombelli@cnr.it (C.B.); 4 Institute for Complex Systems of the Italian National Research Council (ISC-CNR), Sede Sapienza c/o Physics Department, Sapienza University of Rome, Piazzale Aldo Moro 5, 00185 Rome, Italy; simona.sennato@cnr.it

**Keywords:** galactosylated amphiphile, cationic liposomes, *trans*-resveratrol, *Staphylococcus aureus*, MRSA, biofilm, anti-biofilm, bacterial cell wall, anti-adherence effect, antimicrobial resistance

## Abstract

Antibiotic resistance due to bacterial biofilm formation is a major global health concern that makes the search for new therapeutic approaches an urgent need. In this context,, *trans*-resveratrol (RSV), a polyphenolic natural substance, seems to be a good candidate for preventing and eradicating biofilm-associated infections but its mechanism of action is poorly understood. In addition, RSV suffers from low bioavailability and chemical instability in the biological media that make its encapsulation in delivery systems necessary. In this work, the anti-biofilm activity of free RSV was investigated on *Staphylococcus aureus* and, to highlight the possible mechanism of action, we studied the anti-adherence activity and also the cell wall damage on a MRSA strain. Free RSV activity was compared to that of RSV loaded in liposomes, specifically neutral liposomes (L = DOPC/Cholesterol) and cationic liposomes (LG = DOPC/Chol/GLT1) characterized by a galactosylated amphiphile (GLT1) that promotes the interaction with bacteria. The results indicate that RSV loaded in LG has anti-adherence and anti-biofilm activity higher than free RSV. On the other side, free RSV has a higher bacterial-growth-inhibiting effect than encapsulated RSV and it can damage cell walls by creating pores; however, this effect can not prevent bacteria from growing again. This RSV ability may underlie its bacteriostatic activity.

## 1. Introduction

Antibiotic resistance and biofilm-associated infections are currently a global public health problem. Furthermore, some bacterial strains, called “superbugs”, are more of a concern than others because they can be resistant to more than one antibiotic and are able to develop a biofilm. They are responsible for many infections in hospital and community environments and are associated with high mortality rates. Bacteria that hold this sad record include *Staphylococcus aureus* and methicillin-resistant *Staphylococcus aureus* (MRSA) [1]: they are the etiological agents of many infections, including endocarditis, post-operative infections, sepsis, pneumonia, bacteremia, and bone and joint infections [2,3]. The increasingly widespread resistance to conventional antibiotics makes the treatment of these diseases extremely difficult and, thus, there is an urgent need for new drugs or new strategies for effective therapies.

In this regard, bioactive molecules in plants are gaining special attention since plants are constantly exposed to many abiotic and biotic environmental stressors, such as nutrient and water shortages, heavy metals, bacteria, fungi, viruses, and insects; they have faced natural threats for more than 350 million years, co-evolving with their enemies and, thus, developing increasingly effective bioactive compounds to defend themselves [4]. Among them, the phenolic compound *trans*-resveratrol (*trans*-3,5,4′-trihydroxystilbene, RSV) has a wide spectrum of antimicrobial activities, being effective on many Gram-positive and Gram-negative bacterial species. It is a secondary metabolite of plants, produced precisely to fight external pathogen attacks. Notably, RSV is a stilbenoid polyphenol synthesized by seventy-two different plant species and it is particularly abundant in red wine, soy beans, grapes, peanuts, mulberries, blueberries, and pomegranate [5]. In nature, RSV exists in the *trans* form, the most pharmacologically active one, but can be converted into the less active *cis* form by UV-light irradiation or direct sunlight [6].

RSV exhibits antimicrobial and anti-biofilm activity on several *S. aureus* strains, both resistant and non-resistant to diverse antibiotics [5,7,8,9]. It has been suggested that the RSV mechanism of action is related to its ability to interfere with the bacterial cell cycle [8,10] but, to date, the mechanism of bacterial growth inhibition induced by RSV is not yet fully understood [5]. In addition, RSV has the ability to reduce the virulence of *S. aureus* because it decreases hemolysis, reducing the production of α-haemolysin, a protein responsible for both hemolysis and the biofilm formation of *S. aureus* [9,11].

Although it seems well-established that the antimicrobial action of RSV on *S. aureus* is bacteriostatic and not bactericidal, discordant results are reported in the literature, even on the same bacterial strain. In fact, not only are different Minimal Inhibitory Concentration (MIC) values reported but, also, in some experiments, no antibacterial activity is even found. Regarding the anti-biofilm activity, we are faced with the same scenario: some authors report that RSV does not inhibit *S. aureus* biofilm formation [9,11], others report that it reduces the ability of *S. aureus* to form biofilm and that it is able to disrupt mature biofilms [12,13]. Also, in the case of biofilm, it is unclear whether RSV anti-biofilm activity is attributable to its anti-adhesive or bacterial-growth-inhibiting properties. These discrepancies may be due to the different solubility of RSV in the media used for experimental treatments and to the different working culture broths (Mueller–Hinton and Luria–Bertani, respectively). Therefore, conflicting results between studies on RSV susceptibility require further investigation, as recommended in a recent review by M. Vestergaard et al. [5].

Furthermore, it is important to emphasize that, apart from the discrepancies in the *in vitro* data reported in the literature, all the experimental data show RSV antimicrobial properties at concentrations that are higher than those obtainable in plasma following oral administration [14,15,16], as a consequence of the low bioavailability and high biodegradability of RSV. Therefore, there is a limit for the treatment of infections by RSV dictated by the bioavailability of the drug itself [15]. In order to increase RSV bioavailability and, thus, to give a future perspective to the positive results of the *in vitro* studies, it is necessary to turn to nanotechnology, which provides the techniques for developing suitable nanosystems for RSV delivery. In this context, liposomes have been widely shown to improve the antimicrobial efficacy of many drugs against *S. aureus* and MRSA and remain one of the most promising delivery systems, even for combination therapies [1]. To this end, liposomes can be formulated with different kinds of lipids: natural lipids, such as PCs; cholesterol, often used to stabilize the liposome membrane; and synthetic lipids, used to confer liposomes definite properties [17,18,19,20,21]. Therefore, further investigation of RSV-loaded liposomes is a very important topic, particularly for the treatment of *S. aureus* and MRSA infections, on which, to the best of our knowledge, very few scientific articles exist.

In this work, we aimed to contribute to shed light on the effective bacteriostatic and anti-biofilm activity of RSV on two strains of *S. aureus*, wild type (ATCC 25923) and the methicillin-resistant one (or MRSA, ATCC 33591), paying particular attention to the added value brought by liposomal delivery on the antimicrobial properties of RSV. In fact, given the poor solubility of RSV in biological fluids, it is important to study the physico-chemical properties and the actual biological effects of the liposome formulation, which may differ from that of free RSV; in fact, liposomes can enhance or reduce the biological effect or even give rise to a new activity.

**Figure 1:**
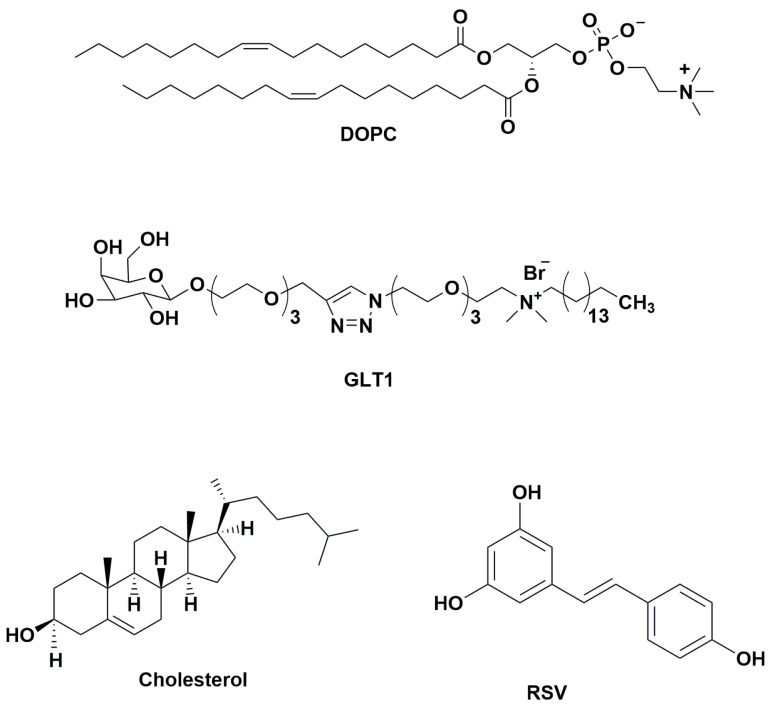
Molecular structures of liposome components (DOPC, Cholesterol, and GLT1) and *trans*-resveratrol (RSV).

RSV was embedded in liposomes formulated with a natural phospholipid (1,2-dioleoyl-*sn*-glycero-3-phosphocholine, DOPC) and cholesterol (Chol), in the presence and in the absence of a cationic galactosylated amphiphile, GLT1, that has been already proven to enhance RSV-loaded liposome activity in the demolition experiments of mature MRSA biofilm [13,22] (Figure 1).

The colloidal stability of the liposomes was monitored over time by Dynamic Light Scattering (DLS) and Dynamic Electrophoretic Light Scattering (DELS) measurements while the interaction between liposomes and both bacterial pathogens was investigated by DELS measurements to reveal any preferential interaction of the galactosylated cationic liposomes with the bacterial surface. The Entrapment Efficiency and the release profile of RSV *in vitro* were evaluated by UPLC analysis.

The anti-biofilm activity of RSV, free and liposome-embedded, was investigated against the *S. aureus* wild type strain, following a mature biofilm experimental protocol assessed in a previous study on MRSA [13]. Furthermore, the anti-biofilm activity was investigated by analyzing the antiadherence effects induced by free or liposome-embedded RSV, against both bacterial pathogens.

Finally, due to the discordant data reported in the literature, we also estimated the bacteriostatic effect induced by free and liposome-embedded RSV on the bacteria strains; moreover, to clarify whether the antimicrobial effect might have been associated with damages to the bacterial cell wall, we stained the bacteria with propidium iodide (PI) in the presence of RSV.

## 2. Materials and Methods

### 2.1. Materials

1,2-dioleoyl-*sn*-glycero-3-phosphocholine (DOPC) was purchased from Avanti Polar Lipids (Alabaster, AL, USA); phosphate-buffered saline (PBS, 150 mM; 10 mM phosphate buffer, 2.7 mM KCl, 137 mM NaCl, pH 7.4, at 25 °C), *trans*-resveratrol (purity 99%), *trans*-stilbene (purity 96%), cholesterol (purity 99%), cellulose dialysis membrane (D9527-100FT, molecular weight cut off = 14 kDa), crystal violet (tris(4-(dimethylamino)phenyl)methylium chloride, purity ≥ 90%), Dulbecco’s Phosphate-Buffered Saline (DPBS, 1×), propidium iodide solution (1.0 mg/mL in water), and chloroform (CHCl_3_, analytical grade) were purchased from Sigma-Aldrich, St. Louis, MO, USA. The galactosylated amphiphile GLT1 was synthesized according to a procedure previously reported [13].

Muller–Hinton (MH) broth and MH agar were purchased from Fisher Scientific (Milan, Italy).

Methanol (MeOH), ethanol (EtOH), acetonitrile (ACN), and water, all HPLC-grade, were purchased from VWR International s.r.l (Milan, Italy); formic acid was supplied by Carlo Erba (Milan, Italy).

### 2.2. Liposome Preparation

Liposomes were formulated with an unsaturated phosphocholine (DOPC) and cholesterol (Chol) in the presence or absence of the cationic galactosylated amphiphile GLT1.

Liposomes, both empty and RSV-loaded, were prepared according to the lipid film hydration protocol, coupled with the freeze-thaw procedure [23], followed by extrusion [24]. Briefly, proper volumes of lipid components dissolved in CHCl_3_ (DOPC and Chol) and MeOH (GLT1) were added in a round bottom flask; the solution was then dried first by a rotary evaporator (Rotavapor R-200, BÜCHI Labortechnik AG, Flawil, Switzerland) and then under high vacuum conditions (5 h) to remove any traces of organic solvents and obtain a thin lipid film. For RSV-loaded liposomes, the proper amount of a RSV solution in absolute ethanol was added to the lipid mixture, before the film formation, to have a molar ratio of 1:8 RSV/lipids. The film was hydrated with 150 mM phosphate buffer saline (PBS) solution to have a final liposomal suspension of 20 mM in lipid concentration. The suspension was vortex-mixed to completely detach the lipid film from the flasks and freeze-thawed five times from liquid nitrogen at up to 50 °C to reduce multilamellarity. RSV-loaded liposomal suspensions were sonicated (probe tip sonicator Sonics Vibra-Cell, 3 mm diameter tip, Sonics & Materials, Newtown, CT, USA) at 40 W (10 cycles of 10 s) to improve their entrapment in the lipid bilayer by breaking aggregates that usually form in aqueous solutions [25]. Size reduction was carried out by the extrusion of liposomal dispersions, ten times (10 mL Lipex Biomembranes, Vancouver, Canada), under high pressure through a 100 nm pore size polycarbonate membrane (Whatman Nucleopore, Clifton, NJ, USA) at a temperature higher than T_m_ to obtain small unilamellar vesicles (SUVs).

Finally, liposome purification from unentrapped RSV was performed by dialysis against PBS under slow magnetic stirring, using a buffer volume 25 times larger than the sample volume.

During all preparation steps (film formation, extrusion, dialysis), samples were protected from light to avoid *trans*-resveratrol isomerization.

### 2.3. Physicochemical Characterization of Liposomes

#### 2.3.1. Size and ζ-Potential

Size distribution, polydispersity index (PDI), and ζ-potential were determined by Dynamic and Dielectrophoretic Light Scattering (DLS, DELS) measurements using a Malvern Nano-Zetasizer analyzer (Malvern Panalytical, Malvern, UK),equipped with a 5 mV He/Ne laser (λ = 632.8 nm) and a thermostatted cell holder. Temperature was fixed at 25 °C in all the measurements.

Particle size and PDI were measured in backscatter detection, at an angle of 173°; this condition represents a significant advantage because it is less sensitive to multiple scattering effects compared to the more conventional 90° configuration and the contribution of micrometric larger particles is considerably reduced [26]. The measured intensity autocorrelation function was analyzed by using the cumulant fit [27]. The first cumulant was used to obtain the apparent diffusion coefficients *D* of the particles, further converted into apparent hydrodynamic diameters, *D_h_*, by using the Stokes–Einstein relationship:

$$D_{h}=\frac{K_{b}T}{3\pi \eta D}$$

where *K_b_T* is the thermal energy and *η* is the solvent viscosity.

The ζ-potential of liposome formulations was determined by DELS measurements. Low voltages have been applied to avoid the risk of Joule heating effects. Analysis of the Doppler shift to determine the electrophoretic mobility was completed by using phase analysis light scattering (PALS) [28], a method that is particularly useful at high ionic strengths, where mobilities are usually low. The mobility μ of the liposomes was converted into ζ-potential using the Smoluchowski relation ζ = μ η/ε, where ε and η are the permittivity and the viscosity of the solution, respectively.

DLS and DELS measurements were performed after the dilution of liposomal suspensions to 1 mM in total lipids in PBS or diluted PBS (15 mM), respectively. Data were collected soon after preparation, after dialysis (for RSV-loaded liposomes), and in the following 14 days (samples stored in the dark at room temperature) in order to evaluate the stability over time and to highlight any aggregation phenomena.

The data reported for hydrodynamic diameter, *D_h_*, PDI, and ζ-potential correspond to the average of at least three different independent experiments.

#### 2.3.2. Determination of RSV Entrapment Efficiency in Liposomes

The content of RSV loaded in liposomes was evaluated by UPLC measurements. Chromatographic analyses were carried out by an Aquity^TM^ UPLC H-Class Bio System (Waters, Milford, MA, USA) equipped with a quaternary pump, a sample manager, an autosampler, a column temperature controller, and a PDA detector.

Before UPLC measurements, samples were properly diluted with methanol to obtain liposome disruption and complete the solubilization of the formulation components. *Trans*-stilbene (TSB) was added (20 μM) to each sample as an internal standard and then all the samples were filtered on PTFE membranes (4 mm × 0.2 μm; Sartorius) before the injection.

RSV determinations were performed on an Aquity UPLC BEH C18 column (50 × 2.1 mm id, 1.7 μm), equipped with an Aquity UPLC BEH C18 pre-column (5 × 2.1 mm id, 1.7 μm). Chromatographic elution was performed in gradient mode, using a mobile phase consisting of 0.1% (*v*/*v*) formic acid in water (phase A), 0.1% (*v*/*v*) formic acid in methanol (phase B), and 0.1% (*v*/*v*) formic acid in acetonitrile (phase C). The gradient program was 0–2 min from 80% A, 10% B, and 10% C to 0% A, 50% B, and 50% C; 2–8.5 min 0% A, 50% B, and 50% C; 8.5–9 min from 0% A, 50% B, and 50% C to 80% A, 10% B, and 10% C. The sample injection volume was 2 µL, the flow rate was 0.25 mL/min, and the column temperature was settled at 40 °C. Both RSV and TSB were detected at 306 nm.

Method validation was determined by assessing linearity, sensitivity, and precision. RSV stock standard solution (305 μM) was prepared in MeOH. Calibration standard solutions in the concentration range between 0.024 and 305 μM (n = 7) were prepared by diluting the stock standard solution of RSV with a methanol–water (80:20, *v*/*v*) mixture. The RSV calibration curve was constructed by triple injections of calibration standard solutions. Each concentration level was spiked with TSB 20 μM. The calibration curve obtained was linear over the concentration range studied, with a correlation coefficient of 0.9995.

The Limit of Detection (LOD) and Limit of Quantitation (LOQ) calculated for RSV were determined by using signal-to-noise ratios of 3 and 10, respectively. The LOD and LOQ were found to be 0.008 μM and 0.024 μM, respectively.

The precision of the method was evaluated in terms of repeatability and reproducibility. Intra- and inter-day precisions, expressed as the relative standard deviation (RSD) of migration time and peak area, were assessed by performing six consecutive injections of the same solution on the same day (RSD < 1%) and over three days (RSD < 2%).

The entrapment efficiency (*EE*%) of RSV in liposomes was calculated using the following Equation (1):

(1) $$EE\;\left(\%\right)=\frac{{\left[RSV\right]}_{pd}}{{\left[RSV\right]}_{0}}\times 100$$

where [*RSV*]*_pd_* indicates RSV concentration after dialysis and [*RSV*]_0_ is the concentration soon after extrusion.

#### 2.3.3. *In Vitro* Release Study of RSV from Liposomes

The release of RSV from the DOPC/Chol and DOPC/Chol/GLT1 liposomes was evaluated by dialysis against PBS (phosphate buffer volume 50 times larger than the sample volume), keeping the systems under constant magnetic stirring. After liposome purification by dialysis, samples were collected every 1 h over a period of 24 h and analyzed by UPLC to study the releasing profile of RSV previously encapsulated. Each liposomal aliquot was diluted with MeOH (1:1 *v*/*v*) to break lipid aggregates and to enhance the release of RSV and the resulting solution was filtered on PTFE membranes (4 mm × 0.2 μm; Sartorius).

In order to determine the percentage of RSV released over the time period, a chromatographic analysis was carried out, as described above, for the EE% determination.

During the whole experiment, samples were protected from light to avoid *trans*-resveratrol isomerization.

### 2.4. Bacterial Strains

The antimicrobial activity of RSV, both free and loaded in liposomes, was examined against two American Type Culture Collection (ATCC) strains of *Staphylococcus aureus*: ATCC 25923 (wild type strain) and ATCC 33591 (methicillin-resistant *Staphylococcus aureus* or MRSA). Both strains were retrieved from the titrated frozen stocks and put in culture in fresh Mueller–Hinton (MH) broth, incubated at 37 °C for 18 h, and then sub-cultured on fresh MH agar plates to have fresh and single colony-forming units (CFUs).

### 2.5. Evaluation of the Liposome–Bacteria Interaction

The binding affinity of neutral and cationic galactosylated liposomes to bacteria cells was investigated by DELS measurements, determining the ζ-potential variation of the solution analyzed.

*S. aureus* and MRSA were grown overnight in MH broth medium; then, they were centrifuged to obtain a bacteria pellet, which was washed 3 times with diluted PBS (15 mM) to remove all traces of culture broth. Afterward, bacteria suspensions in PBS (15 mM) were diluted to reach the desired final density, assessed by optical density (OD) measurements.

Measurements were carried out at 25 °C in 15 mM PBS at different liposome concentrations (0, 0.1, 0.3, 0.5, 0.7, and 1 mM in total lipid concentration) in the presence or absence of 10^5^ CFU/mL of bacteria.

### 2.6. In Vitro Biofilm Formation and Crystal Violet Assay

*S. aureus* was grown in MH medium, supplemented with 0.25 % D-glucose at 1 × 10^7^ CFU/mL by serial dilutions, and submerged biofilm was established in flat-bottom 96-well microtiter plate wells (Thermo Scientific, NUNCLONE-Delta surface, Milan, Italy) at 37 °C for 96 h.

The biofilm formation was measured by Crystal Violet (CV) assay [13]. In brief, wells were washed with DPBS to remove nonattached bacteria and stained with 100 μL of 0.1% CV solution. After 45 min at room temperature, plates were emptied and extensively washed with distilled water to remove the excess CV. For biofilm quantification, 50 μL of 95% ethanol was added to the wells to solubilize all biofilm-associated dye and the absorbance at 530 nm was determined by a microplate reader (VICTOR-NIVOTM, Perkin Elmer, Milan, Italy).

### 2.7. Demolition Assay on S. aureus Biofilm

The biofilm demolition activity of RSV, both free and loaded in neutral and galactosylated liposomes, has been evaluated on the *S. aureus* wild type strain. RSV-loaded liposomes and free RSV (solubilized in DMSO) were sterilized through a 0.22 μm PES filter and diluted with DPBS to have concentrations ranging from 25 μg/mL to 200 μg/mL.

*S. aureus* biofilm was left to grow for 96 h, as described previously. Plates were then incubated at 37 °C overnight with different concentrations of free or liposome-embedded RSV. On the fifth day, wells were emptied, washed with DPBS, and then washed again with DPBS before 100 μL of 0.1% CV solution was added. After 45 min at room temperature, plates were emptied and washed with distilled water to remove excess CV. For biofilm quantification, 50 μL of 95% ethanol was added to the wells to solubilize all biofilm-associated dye and the absorbance at 530 nm was determined by a microplate reader (VICTOR-NIVOTM, Perkin Elmer).

### 2.8. Anti-Adherence Assay

MH agar plates were prepared by spreading 500 μL of the *S. aureus* or MRSA overnight (ON) cultures brought to a density between 1 × 10^8^ and 2 × 10^8^ CFU/mL, by serial dilutions in MH broth, and dried under the sterile flow bench for 15 min. Then, 10 μL of 1.5 µg/mL RSV solution, solubilized in DMSO or loaded in DOPC/Chol/GLT1 liposomes, was dropped on the inoculated agar surface and dried as described above. Furthermore, 10 μL of DMSO and empty liposomes was dropped on control plates. Finally, plates were incubated for 16 h at 37 °C and the anti-adherence activity was evaluated by the naked eye.

For those plates showing a transparent halo, the diameter of the area of transparency was measured to estimate the inhibition of bacteria adherence associated with the investigated sample.

### 2.9. Determination of Minimum Inhibitory Concentration (MIC)

The MIC of the antimicrobials was determined using the standard broth micro-dilution method with slight modifications [29]. Briefly, to assess the MIC of free RSV (solubilized in DMSO) and RSV loaded in DOPC/Chol/GLT1 liposomes, on Day 1, few (5–6) CFUs of fresh *S. aureus* and MRSA plates were grown in 10 mL of MH medium ON at 37 °C. On Day 2, the ON culture was brought to a density of 0.8 × 10^5^–1.2 × 10^6^ bacteria/mL by serial dilutions in MH broth. The final density was estimated by measuring the OD at 600 nm. These diluted cultures were put in a 96-well plate (flat bottom, one plate for each strain studied) where RSV, free or liposome-loaded, was added in triplicates at different concentrations, ranging from 50 µg/mL to 200 µg/mL, and grown ON at 37 °C. The starting bacterial density of the inoculum was verified by plating the final diluted cultures on fresh MH agar and determining the CFUs.

### 2.10. Cell Wall Damage Assay by Propidium Iodide Uptake

*S. aureus* and MRSA strains were put in culture for 16 h in chamber slides (µ-Slide VI 0.4 ibiTreat, 80606, IBIDI GmbH, Gräfelfing, Germany) with free RSV at the respective MIC concentrations identified above (200 µg/mL for *S. aureus* and 100 µg/mL for MRSA). The following day, a propidium iodide solution (1 mg/mL,) was diluted 1:1000 in each chamber slide well to reach a 1 μg/mL final concentration. This dye cannot pass through intact cell membranes but may freely enter cells with compromised cell membranes. After 5 min, the samples were analyzed with an Olympus FV1200 confocal laser scanning microscope with a 20× air objective with an optical pinhole at 1AU and a multiline argon laser at 488 nm, HeNe ion laser at 543 nm, and blue diode laser at 405 nm as excitation sources. Propidium iodide was acquired using excitation at 515 nm and emission at a maximum of 615 nm. Confocal images were processed with ImageJ (National Institutes of Health, NIH, https://imagej.nih.gov/ij; https://imagej.net/software/fiji/ accessed on 29 November 2023).

### 2.11. Statistical Analysis

All statistical analyses were performed with the support of GraphPad prism version 5.0 and Stata software. Data were analyzed by a one-way ANOVA and post hoc Bonferroni comparison tests (*p* < 0.05).

## 3. Results and Discussion

### 3.1. Liposome Preparation

Liposomes as delivery systems of RSV were formulated, as previously described by some of us, with a natural unsaturated phospholipid (DOPC) and cholesterol (Chol) in the presence or in the absence of the cationic galactosylated amphiphile (GLT1) (Figure 1). Moreover, empty liposomes, both neutral and galactosylated were prepared as reference samples.

DOPC was chosen because—on the basis of our experience—a phosphocholine with unsaturated chains allows us to easily obtain monodisperse RSV-loaded liposomes functionalized with amphiphiles [13,30]. As already explained by Aiello et al. [13], cholesterol in the lipid mixture enhances the stability of the lipid bilayer through the bilayer-tightening effect, inducing dense packing and increasing the orientation order of lipid chains. This leads, in general, to a more compact bilayer, with reduced permeability to water-soluble molecules, and increases the retention of entrapped drugs [31]. In this case, in particular, the presence of cholesterol allows the addition of a larger amount of GLT1 to the formulation; in fact, in the absence of cholesterol, large quantities of GLT1 lead to micellar aggregates rather than liposomes [32,33,34]. We underline that the amount of cholesterol present in liposomes (20%) is well below the solubility of cholesterol in DOPC, that is, the threshold at which it is able to form microcrystals within the lipid bilayer [35]. The cationic galactosylated amphiphile GLT1 was designed to enhance the interactions of liposomes with the bacterial cells of *S. aureus* in two ways: on one hand, the cationic charge of the quaternary ammonium group allows an electrostatic interaction with the negatively charged bacterial membrane; on the other, the galactosyl moiety promotes a specific liposome binding to carbohydrate-specific adhesins (i.e., lectins) overexpressed on both bacteria cells and biofilm [36].

For all liposomes, the values of the hydrodynamic diameter (*D_h_*), polydispersity index (PDI), ζ-potential, and RSV Entrapment Efficiency (EE%) were investigated. Results (Table 1) are consistent with those reported by Aiello et al. [13], demonstrating the reproducibility and reliability of the experimental procedures.

### 3.2. Physicochemical Characterization of Liposomes

#### 3.2.1. Stability Studies

**Figure 2:**
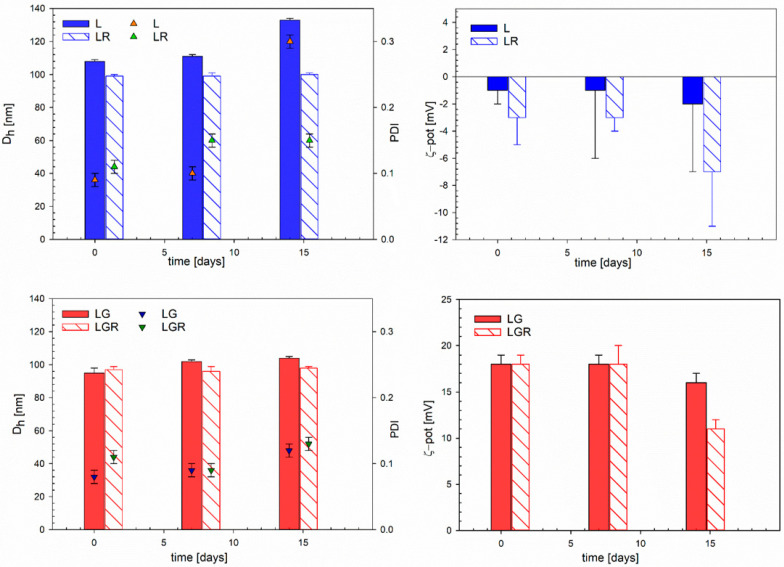
Colloidal properties upon the time of the liposomes (1 mM in total lipids) in the PBS. *D_h_* and PDI are the hydrodynamic diameter and the polydispersity index, respectively, calculated by the cumulants method. Solid bars correspond to RSV-loaded liposomes and stripped bars to the empty ones. The error bars associated with *D_h_*, PDI, and ζ-pot are the standard deviations of three repeated measurements on three different samples.

To investigate the stability over time of neutral and galactosylated liposomes, both empty and RSV-loaded, samples were stored at room temperature and protected from light sources to avoid the isomerization of *trans*-resveratrol to the less active *cis* isomer. The physical stability of liposomes was investigated following the particles’ hydrodynamic diameters, PDIs, and ζ-potential values at different times, up to 14 days of storage. All collected data are reported in Figure 2.

The formulations were stable for two weeks during storage, without any significant change in size and PDI, except for LGR liposomes, for which a decrease in ζ-potential was observed. This effect may be due to the migration of RSV towards the surface, thus towards a more hydrated environment: in these conditions, RSV can partially undergo ionization, with the negatively charged hydroxyl group oriented towards the lipid water interface, thus making the potential less positive [30].

#### 3.2.2. *In Vitro* Release Studies

**Figure 3:**
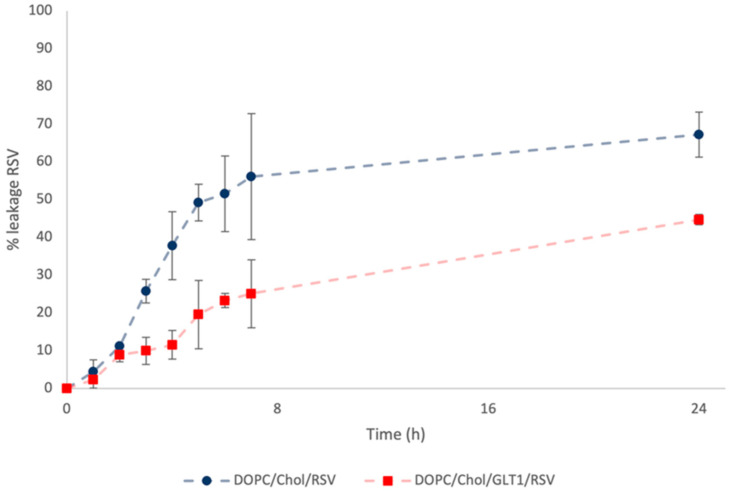
Leakage of RSV up to 24 h from DOPC/Chol liposomes (blue circle) and DOPC/Chol/GLT1 liposomes (red square).

With the goal of evaluating the release profiles of RSV from neutral and galactosylated liposomes, an *in vitro* release study was performed by the dialysis method. Samples were examined by UPLC analysis to determine the % leakage of RSV over time, monitored for a period of 24 h. Figure 3 shows the amount of RSV released from DOPC/Chol and DOPC/Chol/GLT1 liposomes. The % leakage of RSV from DOPC/Chol liposomes was higher than that recorded for galactosylated liposomes. This result could be related to the possible interaction between RSV and the galactosylated amphiphile [37], resulting in a reduction in drug release rate compared to the data collected from the release study of RSV from DOPC/Chol liposomes. However, both release curves highlighted a quite similar trend characterized by a quick release during the first 7 h followed by a gradual slow release up to 24 h. Final RSV leakages of ~60% and ~40% from the DOPC/Chol and DOPC/Chol/GLT1 liposomes were observed, respectively.

#### 3.2.3. Liposomes–Bacteria Interaction

The electrostatic interaction of neutral and cationic galactosylated liposomes with bacteria was investigated by ζ-potential measurements to highlight the formulation’s ability to interact more efficiently with bacteria cells. To this purpose, ζ-potential analysis is considered a simple and powerful tool thanks to the different ζ-potential values that bacteria and liposomes feature, which are determined by both surface charge and ions associated with the surface [38]. Indeed, bacteria always display a negative ζ-potential value while liposomes show variable values according to the lipid components included in the formulation. Neutral liposomes composed of DOPC and Chol are characterized by a slightly negative ζ-potential while cationic galactosylated liposomes composed of DOPC, Chol, and GLT1 show positive values. The interaction between bacteria and liposomes results in different ζ-potential values compared to those of bacteria and liposomes before mixing.

**Figure 4:**
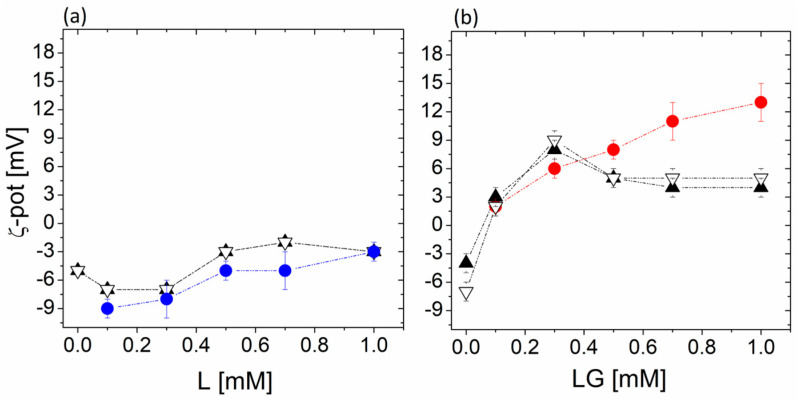
The ζ-potential values of a 10^5^ CFU/mL dispersion of bacteria in PBS 15 mM as a function of added liposome concentration. (**a**) blue circle: L (DOPC/Chol) liposomes alone; white triangle: MRSA in the presence of L liposomes; black triangle: SA in the presence of L liposomes. (**b**) red circle: LG (DOPC/Chol/GLT1) liposomes alone; white triangle: MRSA in the presence of LG liposomes; black triangle: SA in the presence of LG liposomes.

All the experiments were carried out keeping the bacteria density at 10^5^ CFU/mL and varying liposomal concentrations from 0.1 mM to 1 mM in total lipids. In this concentration range, the number of liposomes present in the solution is between 10^11^–10^12^ liposomes/mL [39], always larger than that of bacteria. Thus, in the absence of specific interactions, the ζ-potential value of the mixture will reflect the ζ-potential of the liposomes. The results of the ζ-potential measurements are reported in Figure 4; the ζ-potential values of liposomes alone, as a function of concentration, are also reported as a reference. As expected, both *S. aureus* and MRSA showed a negative zeta potential, around −4 mV and −7 mV, respectively.

After the addition of a low concentration of DOPC/Chol liposomes, the ζ-potential of the mixed liposome–bacteria suspensions is slightly decreased, probably due to the mixing of liposomes and less negative bacteria, both for MRSA and for *S. aureus*. In fact, the behavior of mixed bacteria–liposome suspensions is very similar to that of liposomes alone, thus suggesting that the main contribution to the measured value of ζ-potential stems from neutral liposomes, which do not interact with bacteria. At 1 mM, no difference in ζ-potential can be observed because of the large excess of liposomes in the suspension.

On the contrary, for DOPC/Chol/GLT1 formulation, while in the absence of bacteria, the ζ-potential of the reference liposomal suspension increases with concentration; the different trend measured in the mixed liposome–bacteria suspension suggests the presence of an interaction between bacteria and cationic galactosylated liposomes. The electrostatic interaction between galactosylated liposomes and negatively charged bacteria is evident in the progressive reduction in the measured value of ζ-potential, which reaches a maximum close to 0.3 mM in total lipids then decreases again for both the bacteria strains. Above 0.3 mM, a saturation in the liposome–bacteria interaction probably occurs and liposomes are mostly associated so that a further liposome addition does not promote any variation in the measured value. The observed slight decrease above 0.3 mM could be attributed to the screening effect.

### 3.3. Antimicrobial Properties of RSV

#### 3.3.1. Anti-Biofilm RSV Activity on *S. aureus* Wild Type

**Figure 5:**
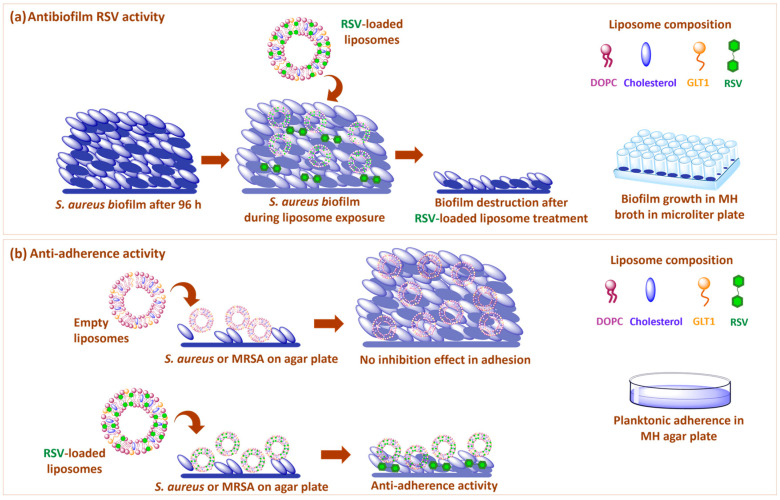
Pictorial representation of RSV-loaded galactosylated liposome activity: (**a**) anti-biofilm activity on the mature biofilm of *S. aureus*; (**b**) anti-adherence activity on *S. aureus* and MRSA.

To assess whether RSV, free or delivered by liposomes, has a different efficacy in reducing biofilm in two different *S. aureus* strains, the wild type or methicillin-resistant one, the biofilm reduction induced by RSV was evaluated on the *S. aureus* wild type strain, following the same protocol described for MRSA biofilm by Aiello et al. [13] (Figure 5a).

Firstly, RSV anti-biofilm activity was evaluated at different concentrations, ranging from 25 μg/mL to 200 μg/mL, determining the biofilm reduction by Crystal Violet assay.

**Figure 6:**
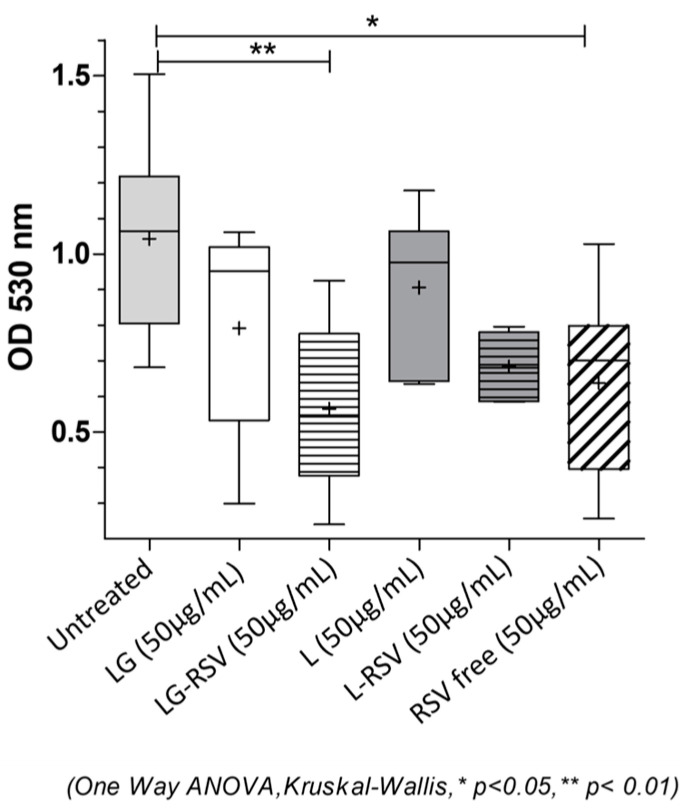
Anti-biofilm activity of RSV, free and liposome-loaded, on *S. aureus* wild type mature biofilm. A statistically significant difference was found only in the comparison between untreated bacteria and LG-RSV-treated ones (** indicates *p* < 0.01) and between untreated and free RSV-treated ones (* indicates *p* < 0.05). + Symbols indicate the mean value of each series.

Figure 6 shows the CV absorbances related to *S. aureus* wild type biofilm demolition in the presence of RSV (free or liposome-embedded) at the concentration that gave the highest biofilm reduction (50 µg/mL). A higher value of absorbance indicates a higher amount of biofilm and, thus, a lower demolition capacity of the system investigated. The CV absorbance obtained for untreated mature biofilm was used as a reference. The experimental data demonstrate that empty liposomes (L and LG formulations) show only a slight reduction in biofilm, which is not statistically significant. In contrast, free RSV induces a biofilm reduction of about 38% compared to that of untreated biofilm (*p* < 0.05), confirming its ability to inhibit biofilm formation.

The ability of free RSV to impair mature *S. aureus* wild type biofilm increases when encapsulated in galactosylated LG liposomes, LG-RSV being more effective than the L-RSV formulation. In fact, LG-RSV liposomes reduce the biofilm formation of *S. aureus* wild type to about half the value of untreated bacteria (representing 54% of untreated cells (*p* < 0.01)) while L-RSV liposomes have an even slightly lower biofilm-reducing capacity (34%, *p* > 0.05) than that of free RSV. These results are consistent with those obtained by S. Aiello et al. [13] for MRSA; additionally, in that case, the galactosylated liposomal formulation LG-RSV displayed a good demolition capacity against mature MRSA biofilm, compared to other liposomal formulations and free RSV.

For this reason, the galactosylated formulation LG-RSV was selected to carry out more in-depth investigations on its antibacterial properties against the two *S. aureus* strains.

#### 3.3.2. Anti-Adherence Activity of RSV on *S. aureus* and MRSA

In order to assess whether the observed anti-biofilm effect could be ascribed to the anti-adherence or bacterial-growth-inhibiting properties of RSV, we performed a Zone Of Inhibition (ZOI) agar-based assay [40] (Figure 5b).

**Figure 7:**
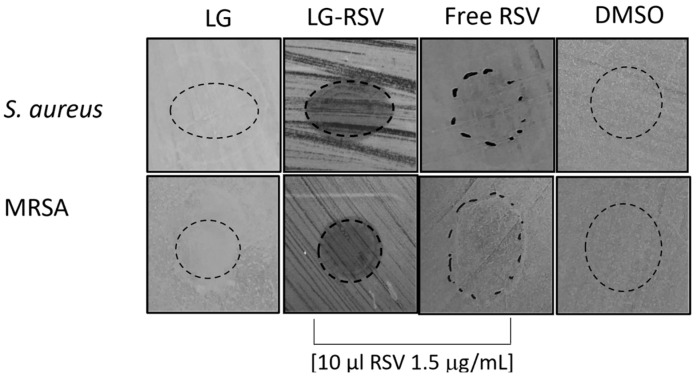
Anti-adherence properties of RSV (free and loaded in liposomes) on *S. aureus* wild type and MRSA bacteria. Empty LG liposomes and DMSO did not induce any visible inhibition halos.

This assay allowed measuring the adherence inhibition halo’s diameter induced by LG-RSV liposomes after overnight bacterial incubation on MH agar plates (see Materials and Methods). Figure 7 shows that LG-RSV liposomes (10 µL per plate of 1.5 µg/mL RSV loaded in LG liposomes) were able to induce a clear anti-adherence effect on both bacterial strains, which was slightly higher in *S. aureus* wild type (mean inhibition halos diameter was 21 mm for *S. aureus* vs. 15.5 mm for MRSA, in two experiments) than in MRSA; on the contrary, free RSV induced only a faint inhibition halo. It is also worth noting that the empty LG liposome did not induce any inhibition in adhesion to the medium agar.

In the literature, it was frequently found that a compound displaying anti-adherence activity is also able to effectively reduce the bacterial biofilm. Such a result was found by many authors with many bacterial species examined and by using natural products [41,42,43]. Therefore, we could figure out that the biofilm initially formed in the presence of RSV encapsulated in LG liposomes might be less stable and more prone to demolition. Additionally, our finding that the treatment with RSV loaded in LG liposomes is also able to reduce an already mature *S. aureus* biofilm, most probably by affecting the bacterial adherence to the substrate, likely enforces this working hypothesis.

#### 3.3.3. MIC Determination of *S. aureus* and MRSA

In light of the results obtained on the biofilm, we aimed to verify whether the encapsulation of RSV could improve not only the anti-adherence capacity but also its inhibiting activity on bacterial growth.

**Figure 8:**
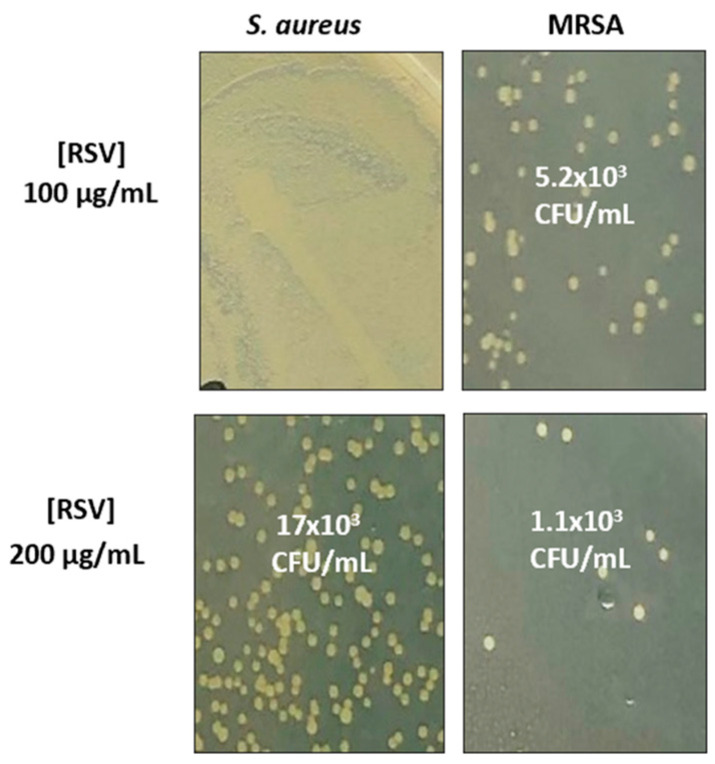
MH agar plates after treatment with RSV. The plates show that the number of MRSA CFUs, after treatment with 100 μg/mL RSV, is roughly three-fold lower (mean CFU count 5.2 × 10^3^/mL) than the correspondent *S. aureus* CFUs (mean CFU count 17 × 10^3^/mL), after treatment with double the amount of RSV (200 μg/mL). The MRSA strain, treated with the latter amount, resulted in roughly 10-fold fewer CFUs (1.1 × 10^3^/mL) compared to *S. aureus*.

In these experiments, unlike previous ones, none of the formulated LG-RSV concentrations assayed induced any inhibiting effect on both bacterial strains’ growth while free RSV displayed bacteriostatic activity on both bacterial strains (Figure 8 shows a representative one of the three experiments performed). This result could be due to the slow release of RSV by LG liposomes in the bacterial culture, resulting in a failure to achieve an effective concentration, as compared with that of free RSV, which is administered all at once in the well.

It is also worth noting that the minimal concentration of free RSV able to inhibit the bacterial growth was lower for MRSA (MIC 100 μg/mL) as compared to *S. aureus* wild type (MIC 200 μg/mL, Table 2).

This different susceptibility to free RSV was confirmed also by the respective CFU counts of the samples that we found inhibited by 200 μg/mL RSV. After another 24 h of growth on MH agar, the CFU count of these transparent wells showed roughly a ratio of 10-fold less MRSA vs. *S. aureus* colonies (Figure 8).

Actually, in our hands, in a very long series of overnight cultures in MH broth, the 600 nm OD of MRSA was constantly lower, by almost 30%, than that of the *S. aureus* wild type. This finding is only apparently surprising because we must carefully consider the overall bacterial fitness of the two *S. aureus* strains. While it is certainly advantageous for MRSA to be *in vivo* resistant to methicillin, in order to survive in hospitalized patients’ infections, it is also true that, in normal growth conditions, its fitness may be lower than the *S. aureus* wild type strain, as observed in our experiments. The selective advantage of bacterial growth under specific stress conditions (like the use of antibiotics, nutrient starvation, the host’s immune response, etc.) could be paid with the toll of a lower fitness in normal conditions of growth. Such a phenomenon has been defined as “fitness cost” and it was described several times for antibiotic resistance in bacteria [44,45].

However, the question remains as to why RSV loaded in LG liposomes inhibits biofilm better than free RSV while it is not able to inhibit the planktonic bacteria like, instead, free RSV does. Biofilms are known to cause chronic infections that are difficult to treat. Most antibiotics are developed and tested against bacteria in the planktonic state, on which they are effective, while they are ineffective against bacterial biofilms. This highlights the importance of investigating the anti-biofilm activity of candidate antibacterial agents, rather than extrapolating from the results of planktonic assays. In our hands RSV loaded in LG liposomes behaves exactly the opposite; it reduces the biofilm of *S. aureus* wild type and MRSA bacteria but does not inhibit their planktonic growth. In order to clarify this finding it will be necessary to deepen the analysis of the cell wall phenotype and the ultrastructure differences existing between planktonic and biofilm staphylococci and focus on the RSV interaction with these two diverse cell wall structures [46,47,48].

#### 3.3.4. Cell Wall Damage Induced by RSV in *S. aureus* and MRSA

The search for new antimicrobials often focuses on the possible damage induced by these compounds on the bacterial cell wall, as documented for many new synthetic and natural products [49,50]. Therefore, we asked whether free RSV might also be able to induce damage to the bacterial cell wall, which would explain its anti-adherence and anti-biofilm effects. To this aim, after overnight incubation with free RSV, *S. aureus* wild type and MRSA were stained with the non-viable dye propidium iodide (PI), which enters only into cells with damaged walls.

According to the results of MIC determination, we treated the MRSA strain with a free RSV solution at half the concentration used for *S. aureus* wild type (100 vs. 200 μg/mL) and analyzed the slide with a confocal microscope.

**Figure 9:**
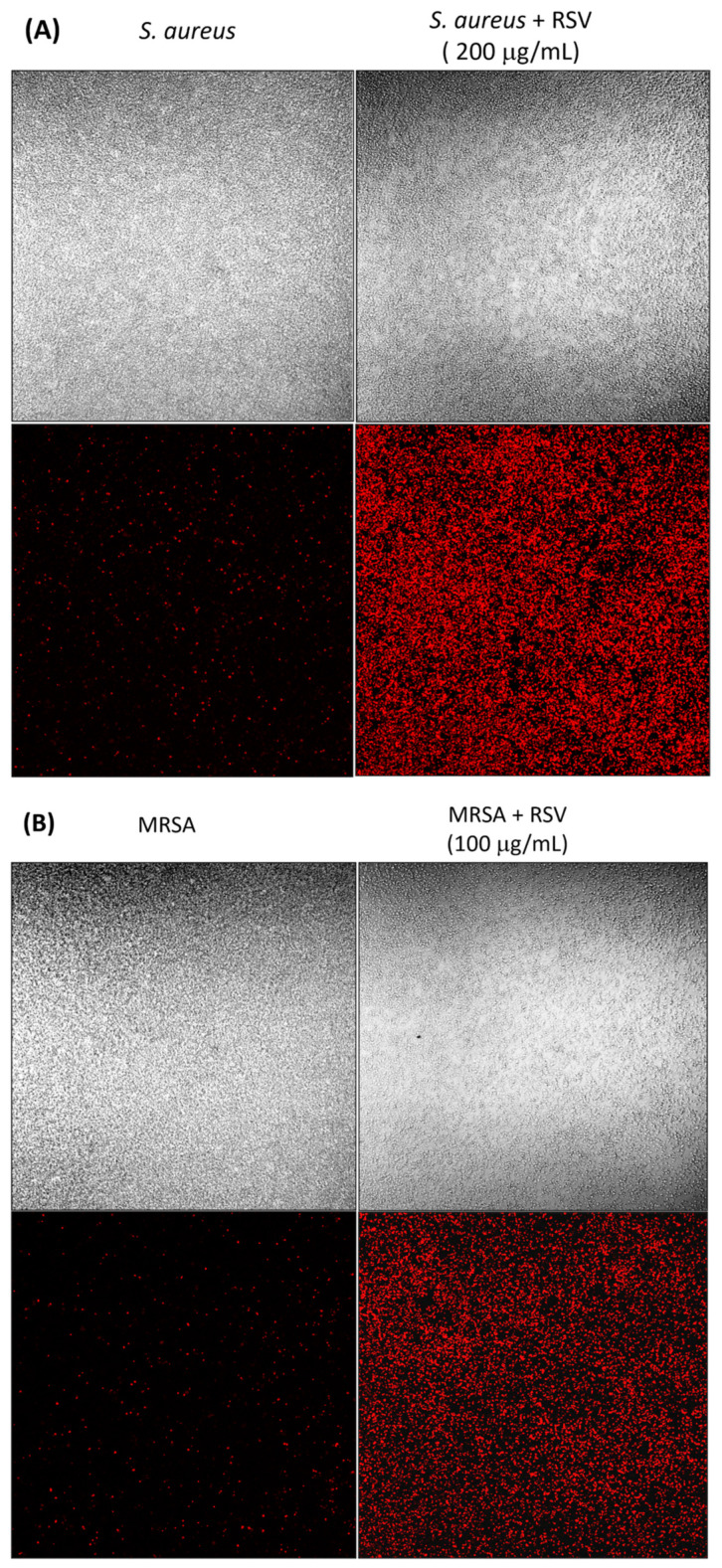
Propidium iodide staining of *S. aureus* wild type (**A**) and MRSA (**B**) bacteria after 16 h incubation with free RSV (200 µg/mL for *S. aureus* wild type and 100 µg/mL for MRSA) to assess the possible cell wall damage induced by RSV. The phase contrast picture (at 40× magnification) is reported in each of the two upper panels while the PI staining is shown in the lower ones.

Figure 9A,B show that RSV-treated bacteria were all positive for PI staining, on the contrary, the untreated controls were positive approximatively for 10–20% of the total bacteria in the wells. This staining possibly says that RSV induces in the bacterial cell wall breaks or pores, through which the fluorochrome may enter.

**Figure 10:**
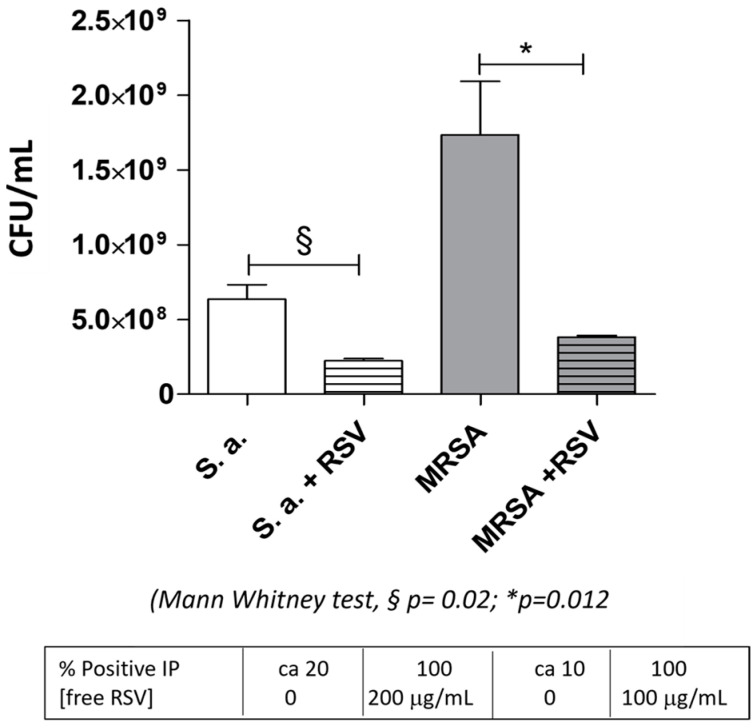
Determination of *S. aureus* wild type and MRSA CFUs after 16 h RSV treatment.

In order to assess whether the positive staining was associated with bacterial cell death, after the microscope analysis, we plated aliquots of the bacteria on fresh MH agar plates. The CFU determination (Figure 10) correctly reflects the bacterial number observed in the relative wells (Figure 9A,B upper panel, phase contrast) of the slide and shows, for *S. aureus* wild type, a roughly three-fold (*p* = 0.02) and, for MRSA, a five-fold (*p* = 0.012) reduction in live bacteria, as compared to the untreated ones.

This is a confirmation of the growth-inhibiting properties of RSV and of the capacity of this stilbenoid polyphenol to affect the bacterial cell wall integrity.

Regarding this result, we are comforted by the extensive literature documenting the ability of propidium iodide to specifically identify the bacterial cells whose membranes have been damaged [51]. Many articles indeed focus precisely on the membrane of *S. aureus* [52].

However, it is worth noting that these cell wall breaks do not prevent RSV-treated bacteria from growing when transferred to fresh medium, possibly being a reversible, or transient, phenomenon. The ability to alter the permeability of the bacterial membrane found for RSV has also been observed for other natural compounds; in fact, Cutro et al. recently observed an increase in the *S. aureus* membrane’s permeability after treatment with an Essential Oil (EO) [50]. The authors show that the EO produces changes in lipid membrane packing, increases the fluidity, and increases the access of water to the interior of the membrane, also affecting the planktonic bacteria. As it is known, EOs contain hundreds of compounds; we are describing the effect of a single component. Nevertheless, further investigation of the *S. aureus* membrane and cell wall after treatment with RSV is required.

## 4. Conclusions

The results presented in this paper are useful building blocks to elucidate the antibacterial activity of RSV, both free and delivered in cationic galactosylated liposomes, on two strains of *S. aureus*, wild type (ATCC 25923) and methicillin-resistant (ATCC 33591). We confirmed that free RSV has weak anti-adhesive activity and good bacteriostatic activity. These results are important for clarifying, as recommended by a recent review [5], the conflicting data reported in the literature about RSV activity. The bacteriostatic and anti-adhesive activities of RSV are presumably related to its ability to form pores in bacteria cell walls. However, the presence of such pores does not impair the ability of bacteria to replicate in the absence of RSV.

We improved with new investigations of the physico-chemical characterization of galactosylated liposomes, empty and RSV-loaded; in particular, we studied the colloidal stability of the formulations and the release of RSV over time. Studying the liposome–bacteria interaction, we pointed out the importance of the galactosylated moiety for the interaction with *S. aureus* and MRSA.

With regard to the biological activity, we demonstrated for the first time that the inclusion of RSV in liposomes functionalized with GLT1 greatly amplifies the anti-adhesive (*S. aureus*, MRSA) and anti-biofilm (*S. aureus*) properties while completely knocking down the bacteriostatic ones. We can reasonably assume that, to exert its antibacterial activity, RSV must interact directly with the wall of the bacteria and that this is not possible if it is included in liposomes. However, given the poor solubility of RSV in biological fluids, we believe that RSV-loaded liposomes are a good candidate as an adjuvant in biofilm-associated antibacterial therapies, thanks to their good anti-adhesive activity.

## Figures and Tables

**Table 1:** Physicochemical features of empty and RSV-loaded liposomes (20 mM total lipids) in PBS (pH 7.4).

Formulation	Composition	*D_h_* ^a^ (nm)	PDI ^a^	ζ-Potential ^a^ (mV)	EE (%)	RSV (mM)
L	DOPC/Chol 8.0:2.0	108 ± 2	0.09 ± 0.01	−1 ± 1	-	-
LR	DOPC/Chol/RSV 8.0:2.0:2.5	99 ± 1	0.11 ± 0.01	−3 ± 2	71 ± 3	1.78 ± 0.08
LG	DOPC/Chol/GLT1 7.5:2.0:0.5	95 ± 3	0.08 ± 0.01	18 ± 1	-	-
LGR	DOPC/Chol/GLT1/RSV 7.5:2.0:0.5:2.5	97 ± 2	0.10 ± 0.01	18 ± 1	88 ± 2	2.11 ± 0.1

^a^ DLS and DELS measurements were performed upon dilution of the samples with PBS 150 mM and 15 mM, respectively, down to 1 mM in total lipids.

**Table 2:** Determination of the Minimum Inhibitory Concentration (MIC) of RSV, both free and loaded in liposomes (LG-RSV), on *S. aureus* wild type and MRSA bacteria. The table summarizes the results obtained after 24 h culture in 96-well plates; the very same aliquots of the transparent wells were put on MH agar plates and, after another 24 h, the bacterial growth was examined.

	MIC Value (μg/mL)	
Bacterial Strain	LG-RSV	RSV
*S. aureus* wild type (ATCC 25923)	n.a.^a^	200
MRSA (ATCC 33591)	n.a.^a^	100

n.a.^a^: no activity.

## Data Availability

Data are contained within the article.
